# Studying the Double Paradox in Air Conditioning at Indian Airports for Airborne Infection Prevention and Filtration of Harmful Suspended Particulate Matter

**DOI:** 10.7759/cureus.23748

**Published:** 2022-04-01

**Authors:** Raja Singh

**Affiliations:** 1 Department of Architecture, School of Planning and Architecture, New Delhi, IND; 2 ISAC Centre for Built Environment Policy, Information Sharing and Analysis Center, Delhi, IND

**Keywords:** suspended particulate matter, dilution ventilation, filtration, airborne infection control, air conditioning systems

## Abstract

Background

Airports are hubs of diverse human interactions. During pandemics, they may serve as centers for the spread of airborne infection. Appropriate methods for the prevention of the spread of airborne infections must be integrated into the air conditioning systems of airports. Along with ultraviolet germicidal irradiation and other sanitization methods, dilution ventilation can be the easiest and most available method for the prevention of airborne infection, which means the intake of outside air into the indoors, which flushes out the aerosolized droplets containing pathogens. Though this process has been adopted by multiple buildings in reaction to the pandemic, it may present the challenge of intake of high concentration of suspended particulate matter in the intake air, a major air pollutant in developing countries, which may enter through the air conditioning systems. Appropriate filtration is necessary so that along with dilution ventilation for airborne disease prevention, the risk of suspended particulate matter of diameter 2.5 micron or PM_2.5_ induced lung issues is also reduced.

Methodology

The Right to Information Act, 2005, was used to file applications for information on the details of the air conditioning systems in Indian airports. The 58 airports in the study were also listed according to the list of cities that fall under the criteria for non-attainment of good air quality standards.

Results

Out of 58 airports considered, 27 fell in the ‘non-attainment’ of good air quality list. On appraisal of filter systems, it was found that 23 had an intake of fresh air but only five had filters with a minimum efficiency reporting value (MERV) of 10 and above in their air conditioning systems, as is recommended for filtration of suspended particulate matter.

Conclusion

It can be concluded that most airports did not have the appropriate filter required for filtering PM_2.5_, which is a major pollutant in Indian cities. In light of coronavirus disease 2019, where dilution ventilation through the intake of outdoor air is suggested, it may also lead to the entry of air with high particulate matter into the indoors.

## Introduction

Airports are hubs of large-scale human interaction. This interaction is all the more critical during the pandemic, when human movement across continents may account for higher chances of spread of infection [1]. Airports are good examples of public spaces where most people are unique visitors. This makes tracking and surveillance of visitors difficult, as obtaining records of previously done activities by these unique visitors is difficult. In the case of airborne diseases, there is a potential risk and unpredictability due to the novel interactions of such people. Particularly in large congregations, which are high-volume enclosed spaces, having airborne infection control measures is, therefore, of utmost importance. At the same time, it is important that dilution ventilation is encouraged, as it leads to a decrease in the concentrations of aerosolized disease-causing organisms like viruses and bacteria [2-6]. Out of the various other airborne infection-control measures, dilution ventilation is particularly suitable due to the easy availability and relative ease of implementation, especially in low-resource settings [6-11]. It basically requires an increased intake of outside air to replace the inside air through higher fresh air changes per hour [12-13]. What is of concern in dilution ventilation is the quality of the air that is allowed to be taken inside [14-16]. This outdoor air should be pollution-free, and, in particular, free from PM_2.5_, which is a key pollutant in cities of the developing world [17-18]. It is also one of the key causes of lung and respiratory issues [19].

This study aims to look at the measures in place in the air conditioning systems of the airports with respect to airborne infection spread. This is to look out for two factors: first, whether there is dilution ventilation (and other measures) for airborne infection control, and second, whether the air conditioning system has the required filtration capacity to filter out PM_2.5_ from the ambient air to be taken indoors [20-21]. This is particularly important for airports that are located in cities with possibilities of high PM_2.5_ concentrations. Airports that are located in the ‘non-attainment list of cities’ under the study were listed and evaluated. The ‘non-attainment list of cities' is a list of cities where the ambient air quality standards prescribed by the Central Pollution Control Board are not met [22]. This included five-year monitoring for ambient air, where if there is an excess of suspended particulate matter (or nitrogen dioxide) as per the National Ambient Air Quality Standards, the cities are listed in the non-attainment list [23-24]. In Indian cities, the major polluter is suspended particulate matter, PM_2.5_, which has an aerodynamic diameter of 2.5 microns [25]. Our study looks at whether the filters present in the air conditioning systems of the airports have the appropriate minimum efficiency reporting value (MERV) capacity, with MERV 10 to 13 and above being ideal to filter out PM_2.5_ [17,26]. This is important to understand because airport managers may be diluting the inside air by allowing in fresh air from the outside [27]. This dilution ventilation plays a key role in flushing out the aerosolized pathogens, but, in this process, also brings in pollutants that the filtration system of the air conditioner must be capable of filtering [4]. Otherwise, owing to a lack of proper filtration, the action of dilution ventilation, appropriate for airborne infection control, may cause harm and lead to lung issues caused due to inhaled particulate matter.

The Airports Authority of India is the statutory body created by an act of the Indian parliament [28]. This body is responsible for creating, upgrading, maintaining, and managing civil aviation infrastructure in India. It also directly manages about 125 airports in India. Being a government-run organization, it is under the purview of the Right to Information Act, 2005 [29]. This Act, aimed at bringing transparency to government functions, is also a boon for researchers who can access information from government bodies and other public authorities. The advantage of this approach is a time-bound, authentic, and ethical source of information that is available in the public domain.

The aim of this study is to perform an appraisal of the air conditioning systems present in Indian airports with respect to the types of air conditioners and the presence of airborne infection control measures. It is to further study the filtration capacity for the inlet air of airport air conditioning systems in cities with considerable levels of ambient pollution, especially with respect to suspended particulate matter PM_2.5_.

## Materials and methods

To undertake the study, multiple steps were involved. The first step was creating a set of questions or parameters for which the information from the airport was required to be received. This was followed by creating a list of airports from where the information needs to be obtained. It was then important to formulate a list of methods that are possible to obtain information from the airports. These methods are listed in Table 1. After deciding on the rationale presented, the suitable method was chosen for obtaining information, i.e., using the Right to Information Act, 2005, for obtaining technical information from the airports [30]. This required follow-up with airports. The information obtained was collated and collected. The information compiled was analyzed against the parameters set in the research. The results were reported and recommendations were made.

**Table 1:** Justification for choosing the methodology Note in point mentioned in serial number 4: The credibility of the information provided under the Right to Information Act, 2005 is derived from the fact that it is covered under the fundamental right to life guaranteed by the constitution of India [30].

S.No.	Method	Advantages and Opportunities	Disadvantages and Threats
1	Seek for secondary data from the airport as they have engineers on board responsible for the air conditioning systems. This will be through correspondence which may be a physical letter or an email.	Getting information directly from the source.	1. Possible reluctance as data sharing may put the airport in a bad light. 2. Lack of accountability in providing a reply, as it depends upon the interest of the airport managers.
2	Visit the airport and seek information by meeting the persons in charge and getting relevant documents.	1. This will enable information direct from the source. 2. Will provide an opportunity to explain the seriousness of the work and its need for research.	1. Visiting the airport may not be the most welcome approach by the airport authorities especially because the building services area may be a no-entry zone due to security prohibitions. The security in Indian airports is managed by the paramilitary with independent set standards of operations, which may not allow for airport visits. 2. Visiting airports during the pandemic, when the study was conducted was especially discouraged due to a higher chance of getting infected in a public space.
3	Visit the airport and perform investigations with respect to the parameters of ventilation and airborne infection control.	1. Most accurate data would be possible to achieve.	1. The process may be bureaucratically daunting and being a high-security area, permission may not be granted. 2. Due to the risk of spread of infection, any person, not part of essential duties, may be prohibited from entering the airport during the pandemic.
4	File an application under the Right to Information Act, 2005 to the airport seeking information and get a reply within the time period stipulated by law.	1. Information right from the source, free from bureaucratic delays as a reply has to be provided within 30 days by law.	1. There may be a bias, but due to the legal nature of the request and the information, the bias is unlikely. The information provided is credible and provided under the seal of the airport's top authorities.

In the study, 90 applications under the Right to Information Act, 2005 were sent to the airports managed by the Airports Authority of India. Out of these 61 replies were received of which 58 have been taken into consideration in this study. The remaining 29 either refused information based on some minor technical discretion or simply did not respond in violation of the Right to Information Act, 2005. Three information replies in the 61 were not fully discernible. This research, therefore, takes into consideration the replies received from 58 airports in India across the five sub-regions of airports in India, namely, northern, eastern, western, southern, and northeastern. Studying 58 airports out of around 129 airports in India represents a substantial number of airports. Because the data provided came from fairly random locations across the nation, made up around 50% of the total airports, and is representative of the various sub-regions of the country, the researchers believe that the sample to meet the criteria for the study may be valid. The sample size has a confidence level of 95% with a margin of error of ±10. This means the sample size is justified. This study was started in the earlier part of the coronavirus disease 2019 pandemic around the year 2020.

The information sought from the airports, mentioned in Table 2, was intended to get a complete picture of the air conditioning systems present in the airport. It was further aimed at obtaining airborne infection control and indoor air quality-related measures in these airports. It was specifically aimed to get pointed information regarding four important points. First, it was to understand whether the airport air conditioning systems had the provision for the intake of fresh air inside the volume of the enclosed space and whether the system provides dilution ventilation for flushing of aerosolized droplets containing pathogens? Second, it was to know the size of the filters used in the HVAC systems of the airport in order to filter the various contaminants like pathogens, dust, and other pollutants like particulate matter. Third, to understand whether ultraviolet germicidal irradiation has been integrated into the air conditioning system of the airport [31]. And fourth, to know whether there are types of air conditioning systems present at the airports, which do not have the capacity to bring in fresh air (or have a reduced capacity) from the outside to the inside. An example would be the split air conditioning system, which recirculates the air inside. The list of cities with non-attainment was directly taken from literature and a comparison was made for the filter sizes and the status of the city with respect to the ambient air quality [22-23].

**Table 2:** The information sought from the airports using the Right to Information Act, 2005 HVAC: heating, ventilation, and air conditioning system

S. No.	Information Requested
1	Type of ventilation and the type of heating, ventilation, and air conditioning system that has been provided in the terminal
2	The steps/precautions taken to prevent the transmission/spread of airborne diseases like coronavirus disease 2019, tuberculosis, measles, etc.
3	Whether there is an integration of ultraviolet light disinfection technologies/ultraviolet germicidal irradiation technology in the heating, ventilation, and air conditioning system present in the interior of the terminal.
4	Whether there are filters used in the HVAC system in the interior of the terminal.
5	The specifications/details of the filters that are being used in the terminal. Please provide the size of the minimum size of particles that can be filtered with the filters used (least count of the filter in microns).
6	Provision of incorporating the outside fresh air into the interior of the terminal via the HVAC system/ventilation system or by any other mechanism.
7	The detail of the fresh air intake capacity of the HVAC/ventilation system used for dilution ventilation, which flushes the inside air and brings in fresh air from the outside.
8	The air changes per hour of fresh air into the terminal.
9	Rooms/spaces in the terminal where split air conditioner/room air conditioners have been used and the inside air is constantly re-circulated.
10	The peak occupancy capacity of the terminal.

Once the required information was received from the airports, it was compiled into tables and a comparison was made among the airports. The analysis was reported and the conclusions were drawn.

This article was previously posted to the engrXiv preprint server on March 12, 2022 [32]. 

Non-requirement of ethics clearance for this study

This study included no questionnaire and no human subject. No employee, visitor, or staff was contacted directly for this study. This study used information available in the public domain through the Right to Information Act, 2005, where an application was made under an appropriate section of the Act and the information was supplied. The information provided was signed and certified by the airport through a senior official and released into the public domain. The Right to Information Act, 2005, allows for the provision of only such information that is not the third-party or personal information of any individual. This prevents any information about any human subject. The use of information available under the public domain and has no human subjects is exempt from review under the 'Indian Council of Medical Research: National Ethics Guidelines for Biomedical and Health Research involving Human Participants' [33]. It further goes on to state that the scope of ethics is only for studies involving human participants, which this study does not involve. According to the above-mentioned scope, this study does not require Ethics Committee Approval or its equivalent Institutional Review Board Approval. The author declares the same.

## Results

There were a total of 58 airports were considered for this study out of 61. Out of these, six did not have flight operations but had an operational building. The type of air conditioner system in the airports varied from being full-fledged central heating, ventilation, and air conditioning system to ones with split air conditioners in a room. The total diversity in air conditioning systems is given in Table 3. The filtration capacity of the air conditioning systems of the airports has been listed in Table 4. Out of the 58 airports, 43 had a filtration of some kind, the majority being pre-filters of the MERV value or minimum efficiency reporting values in the range of 5 to 8. Only five had filters of MERV value equal to or above 10. MERV is the unit in by which the filtration capacity of filters is measured. A high MERV value means the filter can arrest finer particles. Studies have shown that MERV 10 and above are more capable of arresting particulate matter PM_2.5_. It was important to understand the ambient air quality of the cities where the airports were located. The Central Pollution Control Board of the Indian government has listed cities that are in the 'non-attainment category'. This means that the ambient air quality does not meet the air quality standards. In India, the primary ambient air pollutant is PM_2.5_. It was found that out of the cities where the airports under the study were located, 27 cities came under the non-attainment category. Out of these, 23 had an intake of fresh air through the HVAC system, but only five had filters of rating MERV 10 and above in their air conditioning systems. The consequences of these values have been further discussed in the discussion section.

**Table 3:** List of the type of air conditioning systems along with the numbers of airports having these systems OEM: original equipment manufacturer; HEPA: high-efficiency particulate absorbing filter; MERV: minimum efficiency reporting value; HVAC: heating, ventilation and air conditioning; VRV/VRF: variable refrigerant volume/variable refrigerant flow; HVLS: high velocity low speed

S. No.	Type of air conditioning system	Total number of airports
1.	Central HVAC system	18
2.	VRV/VRF/package/cassette type	8
3.	Split/ room air conditioner/ tower type standalone	12
4.	HVAC + split	8
5.	HVAC + VRV	1
6.	Heat generator system	1
7.	Details not provided	3
8.	HVAC + HVLS fan	1
9.	No HVAC	4
10.	VRF + split combination	2

**Table 4:** List of the filtration capacity of filters integrated into the air conditioning systems of the airports studied MERV: minimum efficiency reporting value; HEPA: high-efficiency particulate absorbing filter; HVAC: heating, ventilation, and air conditioning

S. No.	Filtration capacity of filters integrated into the HVAC system of the airports	No. of airports with the mentioned filtration capacity
1	MERV <5-6	5
2	MERV 5-6	2
3	MERV <7-8	8
4	MERV 7-8	6
5	MERV 10	1
6	MERV 8	2
7	MERV 13	1
8	MERV 8 + MERV 14	1
9	MERV 8 + MERV 11	1
10	HEPA	2

Six out of 58 had installed an ultraviolet germicidal irradiation system while 19 had the work of installation in the pipeline at the time of the study. Forty-three out of the 58 had provision for a filter within the HVAC system. The filter type details are listed in Table 4. A separate list was made of airports that fall in cities that come under the non-attainment list of ambient air quality by the Central Pollution Control Board of India [22]. Table 5 contains the list of the airports that fall in the cities with non-attainment. It was found that out of the 58, 27 came under the non-attainment category. Out of these, 23 had an intake of fresh air through the HVAC system, but only five had filters of rating MERV 10 and above in their air conditioning systems.

**Table 5:** The appraisal of the air conditioning systems, fresh air intake capability, and filtration capacity of airports located in cities on the non-attainment list of ambient air quality H: centralized HVAC system present; V: VRF/VRV/cassette/package type air conditioning system present; S: split/tower type standalone air conditioning system present; n.p: information not provided; OEM: original equipment manufacturer; HEPA: high-efficiency particulate filter; MERV: minimum efficiency reporting value; HVAC: heating, ventilation and air conditioning; VRV/VRF: variable refrigerant volume/variable refrigerant flow

S. No.	List of cities where the airports are located and which fall under the non-attainment list of ambient air quality.	Type of air conditioning system (see legend)	Whether fresh air through HVAC is supplied. (1: Yes; 0: No)	Rating of the filtration capacity of filters integrated into the HVAC (value in MERV)
1	Udaipur, Rajasthan, India	H	1	8
2	Gwalior, Madhya Pradesh, India	V	1	5-6
3	Kanpur. Uttar Pradesh, India	V+S	0	n.p
4	Ludhiana, Punjab, India	S	0 (n.p)	5-6
5	Srinagar, Union territory of Jammu and Kashmir, India	H+S	1	6-8
6	Agra, Uttar Pradesh, India	H+S	1	8
7	Allahabad (Prayagraj), Uttar Pradesh, India	H	1	8+11
8	Dehradun, Uttarakhand, India	H	1	<7-8
9	Ghaziabad, Uttar Pradesh, India	V	1	8
10	Jammu, Union territory of Jammu and Kashmir, India	n.p	1	n.p
11	Amritsar, Punjab, India	H	1	>14
12	Mohali/Chandigarh, Punjab/union territory of Chandigarh, India	H	1	13
13	Patna, Bihar, India	H+V	1	13
14	Raipur, Chhattisgarh, India	H	1	10
15	Bhubaneshwar, Odisha, India	H+S	1	<7-8
16	Gaya, Bihar, India	H+S	1	<5-6
17	Silchar, Assam, India	S	0	OEM(HEPA)
18	Dimapur, Nagaland, India	V	1	n.p
19	Pune, Maharashtra, India	H	1	5-6
20	Vadodara, Gujarat, India	H	1	<7-8
21	Akola, Maharashtra, India	S	0	n.p
22	Aurangabad, Maharashtra, India	H	1	<7-8
23	Indore, Madhya Pradesh, India	H+S	1	<7-8
24	Jalgaon, Maharashtra, India	S	0	n.p
25	Visakhapatnam, Andhra Pradesh, India	H	1	<5-6
26	Pathankot, Punjab, India	H	1	<7-8
27	Kolkata, West Bengal, India	H	1	8

## Discussion

The study brought to light the state of the air conditioning systems at airports in India. Airports, being a primary source of diverse human interaction, pose a key threat to the spread of airborne infections. Appropriate methods for airborne infection control are recommended to reduce the infection spread. The study found that out of the 58 airports in question, only 18 had a full-fledged heating, ventilation, and air conditioning system with the presence of air-handling units for fresh air intake or the possibility of ultraviolet germicidal integration [27,31,34]. Using ultraviolet germicidal irradiation has been suggested, but there may be concerns regarding its use [35]. Out of the remaining, 12 were depending on split air conditioning systems, which have no provisions for fresh air intake and constantly recirculate the air inside the space [9]. These spaces cannot provide a predetermined and measurable quantity of fresh air into the space and have to depend upon measures like opening the windows and gates in some cases [12]. There were others that used a package-type air conditioner system using variable refrigeration flow. This technology is used due to the fact that it achieves energy savings due to lower demand-based usage. The downside to this technology is the absence of a full-fledged air handling unit. In some cases, treated air units were installed, but it may lack the possibility of quantification that air handling units can achieve through calculated customization. Talking about the integration of ultraviolet germicidal irradiation in the air conditioning system, out of the 58 airports, six had installed the UVGI system at an earlier stage of the pandemic, when the study was conducted while 19 others were in the process of installation of the ultraviolet systems.

Talking about the filtration capabilities to allow for fresh air intake, out of 58, 43 had filtration of some kind, the majority being pre-filters of MERV values in the range of 5 to 8. Only five had filters of MERV value equal to or above 10. This means their general capability to filter PM_2.5_, the major pollutant in most cities of the developing world, is very low. This is because studies have shown that for the filtration of fine suspended particulate matter, MERV 10 and above is suitable [17]. It is very important that the fresh air intake in the cities that fall in the ‘non-attainment’ list of ambient air quality in India be filtered and made free from PM_2.5_, which is the major pollutant, caused due to burning of fossil fuels like coal, petrochemicals, and farm waste. Out of the 58 airports, 27 were located in cities that were in the non-attainment category. This means that the ambient air quality of these cities did not meet the national standards of the recommended levels of pollutants in ambient air. In India, one of the primary pollutants is PM_2.5_, which is the leading cause of lung diseases [19,36]. Out of these 27 airports, 23 had an intake of fresh air through the HVAC system, but only five had filters rating MERV 10 and above in their air conditioning systems. This means that the air intake in these airports for airborne infection control may lead to an increased air pollutant concentration inside, leading to the situation of a double paradox, where the air intake is important for airborne infection control but may consequently lead to bad indoor air quality due to high pollutant concentrations. This hints at a lack of preparedness for the particulate matter pollution that is a leading cause of lung issues in India [19].

This is all the more critical when the right to a healthy environment has time and again been declared a human right [37]. Efforts need to be put in place to have appropriate measures for indoor air quality in large public spaces. There should be constant monitoring and the levels of the indoor air quality must be displayed to the occupants of the space so that awareness about this issue can be created [14]. Ventilation of buildings needs more focus and this is not a mere engineering problem but a public health issue deserving urgent attention [38-39].

## Conclusions

As stated in the discussion section, the airports studied may have a further scope of work to be done to improve their air conditioning systems for suitable indoor air quality. This is not only to prevent the spread of airborne infections but also to filter out the PM_2.5_ that may enter through the inlet of the HVAC systems in these airports, and cause respiratory issues for the occupants. The situation of this double paradox exists for 27 airports that may have to increase fresh air intake for dilution ventilation for infection control, but out of these, only five had filters that could efficiently filter most of the PM _2.5_. This means that the air intake may not only lead to dilution ventilation but may also increase the concentration of PM_2.5_ in indoor spaces. This indicates that large public buildings in India, especially airports, which are places of high stranger exposure, may need steps to ensure appropriate airborne infection control measures and appropriate indoor air quality. This can be achieved by having well-thought-out laws governing indoor air quality and the allied equipment made with inputs from a wide range of disciplines, including medicine, engineering, architecture, social sciences, epidemiology, and public health. The enforcement of indoor air quality standards must be strictly performed and must be placed under legal obligation, and not just be mere guidelines. An appropriate way of doing so may be by including indoor air within the purview of air pollution laws in countries across the world. With this action, indoor spaces of large congregate spaces will come under regulation. The buildings must be regularly audited from the airborne infection control and indoor air quality point of view. This is all the more relevant when we are in the current universal indoor air crisis, facing the threat of airborne pandemics and an allied problem of particulate matter pollution in cities of the developing world. Indoor air quality must be treated as a public health issue and not merely an engineering problem.
